# *Boechera* or not? Genomic insights and taxonomic reassessment of the misclassified Asian species *B. calcarea* (Brassicaceae)

**DOI:** 10.1016/j.pld.2025.06.009

**Published:** 2025-07-10

**Authors:** Terezie Mandáková, Milan Pouch, Petra Hloušková, Dmitry A. German, Pavel Trávníček, Michael D. Windham, Martin A. Lysak

**Affiliations:** a Central European Institute of Technology, Masaryk University, Brno, Czech Republic; b Department of Experimental Biology, Faculty of Science, Masaryk University, Brno, Czech Republic; c National Centre for Biomolecular Research, Faculty of Science, Masaryk University, Brno, Czech Republic; d South-Siberian Botanical Garden, Altai State University, Barnaul, Russia; e Institute of Botany, Czech Academy of Sciences, Průhonice, Czech Republic; f Department of Biology, Duke University, Durham, USA

**Keywords:** Arabideae, Cruciferae, Chromosome structure, Centromere repositioning, Cytogenetics, Phylogenetic reconstruction

## Abstract

The genus *Boechera* (Brassicaceae) serves as a model system for studying apomictic reproduction and ecological adaptations, with most species occurring in North America. The rare occurrence of *Boechera* species outside their typical range provides unique opportunities to investigate genome evolution in extralimital environments. One such species, *B. calcarea*, has been described from the Chandalaz Mountains in northeastern Asia (Russia). This study aimed to investigate the genome structure and evolutionary history of *B. calcarea*. However, our analyses reveal that the species does not belong to *Boechera*. Instead, an integrative approach combining cytogenetic, phylogenetic and repeatome analysis identified the species as a member of one of more ancestral clades in the tribe Arabideae. The diploid *Parryodes calcarea* (2*n* = 16) exhibits Arabideae-specific chromosomal signatures, including multiple centromere repositionings. These findings clarify the misclassification of *P. calcarea* as *Boechera*, leaving *Boechera falcata* and *Borodinia macrophylla* as the only representatives of the Boechereae in the Old World. This study highlights the importance of an integrative approach in resolving taxonomic ambiguities and provides new insights into the diversification of the largest cruciferous tribe, the Arabideae.

## Highlights

• Cytogenetic, phylogenetic and repeatome analyses identified the new species formerly described as *Boechera calcarea* (Brassicaceae).
• The new species, *Parryodes calcarea*, is a member of the tribe Arabideae
• Importance of integrative approach in resolving taxonomic ambiguities.
• New insights into the diversification of the largest cruciferous tribe.

## 1 Introduction

The classification of plants into genera and species is fundamental to botany, offering a framework for understanding biodiversity, ecological adaptations and the evolutionary history of life on earth. Traditionally, plant taxonomy has relied heavily on morphological characters such as fruit shape, trichome morphology and reproductive traits to group species into genera. While these phenotypic markers are convenient, they often do not reflect true evolutionary relationships. Convergent evolution can obscure phylogenetic signals and lead to misclassifications (Smith et al., 2020). Recent advances in molecular phylogenetics and cytogenetics have revolutionized the field, providing botanists with powerful tools to unravel evolutionary history and reassess taxonomic boundaries, especially in plant families with frequent morphological convergence (e.g., Mandáková et al., 2019; Guo et al., 2021; Mandáková and Lysak, 2022; Hendriks et al., 2023; Al-Shehbaz, 2025).

The family Brassicaceae, comprising approx. 4000 species in 349 genera, ranks among the largest and most ecologically diverse plant families (e.g., Hendriks et al., 2023; Al-Shehbaz, 2025). Its species exhibit remarkable morphological, physiological and ecological variability, which contributes to the taxonomic complexity of the family. Within the Brassicaceae, the tribe Arabideae has presented persistent challenges in earlier classifications due to its morphological overlaps and polyphyly. In the past, classifications relied on traits such as latiseptate siliques, branched trichomes and accumbent cotyledons (Hopkins, 1937; Rollins, 1941). However, molecular and cytogenetic studies showed that the traditional circumscription of *Arabis* was polyphyletic and included several distinct evolutionary lineages. These findings necessitated numerous transfers of former Arabideae species to genera outside the tribe, such as *Arabidopsis*, *Boechera*, *Catolobus*, *Pennellia* and *Turritis* (Koch et al., 1999, 2001; 2003; 2012; Al-Shehbaz, 2003, 2005; Warwick and Sauder, 2005; Al-Shehbaz et al., 2006; Kiefer et al., 2009; Hendriks et al., 2023).

The genus *Boechera* (Boechereae), which was separated from *Arabis* by Löve and Löve (1976), has since been confirmed as a monophyletic group based on molecular and cytogenetic evidence (Al-Shehbaz, 2003). *Boechera* is characterized by its base chromosome number of *x* = 7, in contrast to *x* = 8 in *Arabis* s.str., as well as by its unique reproductive biology, including the production of asexual seeds via diverse developmental pathways (Carman et al., 2019) and the widespread occurrence of diploid apomixis (Al-Shehbaz, 2003; Koch et al., 2001; Beck et al., 2012; Mandáková et al., 2020a). Morphologically, *Boechera* is characterized by dendritic, irregularly bifurcate or sessile leaf trichomes, often bent and/or pendent fruits and some other features that are absent or rare in *Arabis* (Al-Shehbaz, 2003). These traits, together with molecular and cytological evidence, clearly distinguish the two genera and tribes.

While *Boechera* occurs primarily in North America, particularly in the western United States, the discovery of species outside the continent provides valuable insights into the evolutionary history and dispersal mechanisms of the genus. These extralimital taxa challenge the notion that *Boechera* is a strictly North American lineage and suggest historical biogeographic connections between the continents (Al-Shehbaz, 2003; Windham and Al-Shehbaz, 2006, 2007). Such species may have arisen through long-distance dispersal or as remnants of a broader ancestral distribution facilitated by climatic and geological phenomena such as the Bering Land Bridge during glacial periods (Abbott et al., 2000).

The most extensively studied extralimital Boechereae species is *Boechera falcata* (Zilov et al., 2025), which occurs in Siberia and the Russian Far East. The morphological similarity of *B. falcata* with its North American relatives (e. g., with *B. holboellii*, the type of *Boechera*: Busch, 1926; Schulz, 1936; Berkutenko and Gurzenkov, 1976, in all cases given as *Arabis*) was noticed long ago, and its inclusion in the genus was subsequently supported by phylogenetic studies (Kiefer et al., 2009, 2014). Cytogenetic analysis revealed a diploid chromosome number of 2*n* = 14, which is consistent with the genus *Boechera* and further supports its phylogenetic placement (Yurtsev and Zhukova, 1972; Berkutenko and Gurzenkov, 1976). Another species of particular interest is *B. calcarea* described by Doudkin and Volkova (2013) from the Chandalaz mountain Range in the Primorsky Territory, Russia. According to the authors, *B. calcarea* was characterized as an obligate calciphile thriving on nutrient-poor calcareous soils and differs morphologically from *B. falcata* by larger fruits, forked (as opposed to substellate) trichomes and pale lavender (vs. pink) petals (Fig. S1A). *B. calcarea* was reported to have a diploid chromosome number of 2*n* = 14 (Doudkin and Volkova, 2013; Fig. S1B), which is consistent with the dominant chromosome number in *Boechera* (Mandáková et al., 2020a). Unlike *B. falcata* (Zilov et al., 2025), *B. calcarea* has not been extensively studied in terms of its genetic structure, phylogenetic position or reproductive biology, leaving significant gaps in our understanding of its evolutionary history.

Here we present a comprehensive genomic characterization of *Boechera calcarea* with the aim of investigating the genome structure and evolutionary history of *B. calcarea*. Surprisingly, our analyses have shown that the species belongs neither to *Boechera* nor to any other genus of the tribe Boechereae. Instead, it is reclassified as *Parryodes calcarea* (Dudkin) D.A. German & Lysak (Chepinoga et al., 2024) and belongs to one of more ancestral clades in the tribe Arabideae.

## 2 Materials and methods

### 2.1 Plant material

**Fig. 1:**
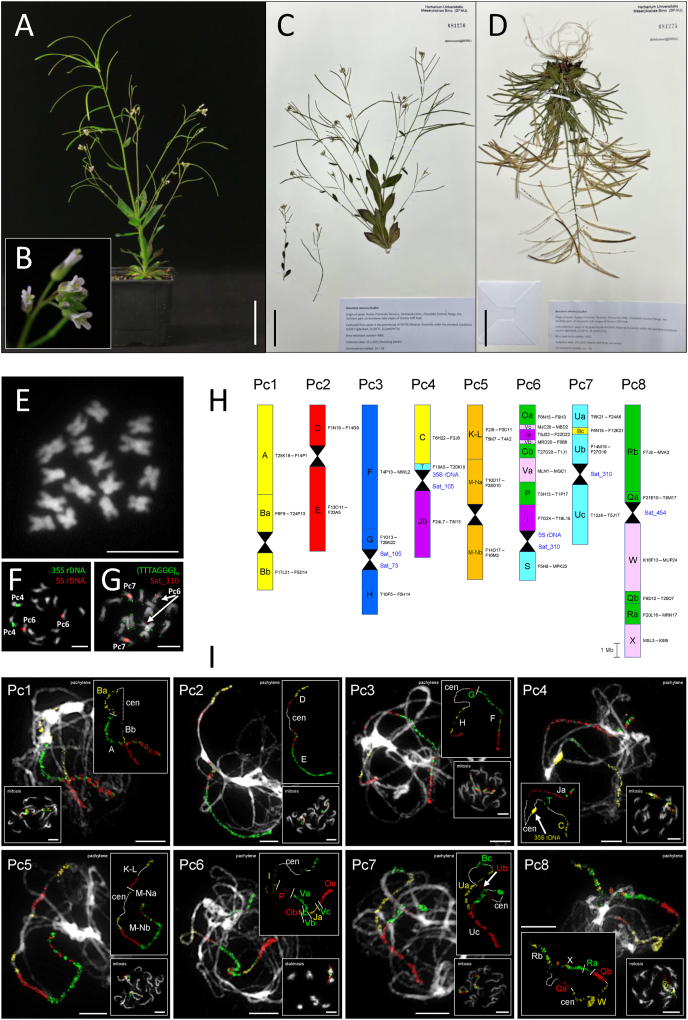
Genome structure of *Parryodes calcarea*. (**A**) Photograph of cultivated plant. (**B**) Close-up of inflorescence. (**C**) Herbarium specimen of flowering plant accession investigated, deposited in the herbarium of Masaryk University (BRNU). (**D**) Herbarium specimen of plant bearing fruits. (**E**) Mitotic chromosome spread prepared from a root tip, showing 2*n* = 16 chromosomes. (**F**) Mitotic chromosomes hybridized with 35S and 5S rDNA probes. (**G**) Mitotic chromosomes hybridized with telomeric and centromeric satellite Sat_310 probes. (**H**) Karyotype structure inferred from comparative chromosome painting (CCP), with the eight individual chromosomes labelled as Pc1–Pc8. Capital letters (A to X) represent the 22 genomic blocks (GBs) of the Ancestral Crucifer Karyotype (ACK; Mandáková et al., 2019), some of which are reshuffled into sub-blocks (e.g., Ba, Bb, Bc). Each GB is color-coded to correspond to its original position on the eight chromosomes of the ACK. Hourglass symbols indicate centromeres, and Arabidopsis BAC clones are provided as markers for each (sub-)block. Chromosomes are drawn to scale (scale bar = 1 Mb). (**I**) The eight chromosomes (Pc1–Pc8) were visualized through CCP using Arabidopsis BAC contigs as painting probes applied to pachytene, mitotic, and diakinesis chromosome spreads. Painting signals are shown in experimental colors, reflecting the fluorochromes used to label specific GBs. When a GB is labelled with a single fluorochrome, its letter is displayed in the corresponding color. For subdivided GBs labelled with multiple fluorescent dyes to indicate their orientation, the letters are shown in white. Centromeres are marked as “cen”. All chromosomes were counterstained with DAPI. Scale bars are 5 cm for plant photographs (A, C, D) and 10 μm for chromosome images (E, F, G, I).

Seeds were collected by Roman V. Doudkin on exactly the same locality from which *Boechera calcarea* was described (Doudkin and Volkova, 2013): Russia, Primorsky Territory, Partizansky Distr., Chandalaz (Lozovy) Range, the northern part, on limestone rocky slopes of Chertov Cliff Peak. Plants were cultivated from seeds in the greenhouse of CEITEC Masaryk University under the standard conditions (16/8 h light/dark, 21/18 °C, 15 μmol/m^2^/s) (Fig. 1A and B). Specimens are deposited at the Herbarium of Masaryk University (BRNU), sheet numbers 681276 (flowering plant; Fig. 1C; https://brnu.jacq.org/BRNU681276) and 681275 (plant with fruits and seeds; Fig. 1D; https://brnu.jacq.org/BRNU681275).

### 2.2 Chromosome preparation

Actively growing root tips were harvested from cultivated plants, pre-treated with ice-cold water for 12 h, and subsequently fixed in a freshly prepared ethanol:acetic acid solution (3:1) for 24 h at 4 °C. The fixed samples were then stored at −20 °C for future use. Entire inflorescences were similarly fixed overnight in freshly prepared ethanol:acetic acid solution (3:1) and stored at −20 °C until processing. Mitotic and meiotic (pachytene) chromosome preparations were conducted following the protocol described by Mandáková and Lysak (2016a).

### 2.3 Probe preparation

A total of 674 chromosome-specific BAC clones from *Arabidopsis thaliana* (Arabidopsis) were used, grouped into contigs based on 22 genomic blocks as described by Mandáková et al. (2019). Painting probes were designed according to the known genome structure of *Pseudoturritis turrita* and other Arabideae crown-group species (Mandáková et al., 2020b; Nowak et al., 2021). To achieve detailed chromosome structure characterization and precise localization of centromeres, initial experiments were followed by fine-scale painting using shorter BAC (sub)contigs. Individual, differentially labelled BAC clones were applied for BAC-by-BAC centromere delimitation. For fine-scale centromere mapping, BAC contigs were selected based on their known position within genomic blocks flanking or containing centromeric regions in the Arabideae genomes (Mandáková et al., 2020b). These contigs were selected to provide high-resolution coverage across the expected centromeric intervals, with priority given to clones spanning the transition zones between pericentromeric heterochromatin and adjacent euchromatic regions. The selection was further refined by iterative hybridization experiments to confirm signal specificity and centromere positions. The localization of 5S and 35S rDNA loci was achieved using clone pCT 4.2 (corresponding to the 500-bp 5S rRNA repeat, GenBank accession M65137) and Arabidopsis BAC clone T15P10 (GenBank accession AF167571), respectively. An Arabidopsis-like telomeric probe was prepared using the protocol outlined by Ijdo et al. (1991). Additionally, five species-specific tandem repeats (Sat_73, Sat_105, Sat_177, Sat_310 and Sat_454) were identified and prepared as described below and following Dogan et al. (2021). All probes were labelled with biotin-dUTP, digoxigenin-dUTP or Cy3-dUTP via nick translation, then pooled, precipitated and resuspended in 20 μl of hybridization mixture (50% formamide and 10% dextran sulfate in 2 × SSC) per slide, following the published protocol (Mandáková and Lysak, 2016b).

### 2.4 Comparative chromosome painting and in situ *hybridization of repeats*

Prior to the analysis, suitable slides were pre-treated with RNase (100 mg/ml) and pepsin (0.1 mg/ml). Chromosome and probe denaturation was performed simultaneously on a hot plate at 80 °C for 2 min, followed by overnight incubation in a humidified chamber at 37 °C. Post-hybridization washes were carried out in 20% formamide in 2 × SSC at 42 °C. Fluorescent detection was conducted according to Mandáková and Lysak (2016b) as follows: biotin-dUTP was detected using avidin–Texas red, with signal amplification by goat anti-avidin–biotin and avidin–Texas red; digoxigenin-dUTP was detected using mouse anti-digoxigenin followed by goat anti-mouse Alexa Fluor 488. Chromosomes were counterstained with 4′,6-diamidino-2-phenylindole (DAPI; 2 μg/ml) in Vectashield.

### 2.5 Image processing

Fluorescent signals were analyzed and photographed using a Axioimager epifluorescence microscope (Zeiss) and a CoolCube camera (MetaSystems). Images were acquired individually for the four fluorochromes using corresponding excitation and emission filters (AHF Analysentechnik). The captured images were pseudocolored and merged using Adobe Photoshop CS6 software (Adobe Systems). Circular visualizations of chromosome-scale pseudomolecules were prepared using Circos (Krzywinski et al., 2009).

### 2.6 Phylogenetic analysis

Genomic DNA was extracted from young leaves using the NucleoSpin Plant II kit (Macherey–Nagel). Internal transcribed spacers (ITS1 and ITS2) were amplified using the primer pair ITS1/ITS4 (White et al., 1990), and the purified amplicons were Sanger sequenced at Macrogen and submitted to GenBank (PV061832). These sequences were then analyzed using BrassiBase (Kiefer et al., 2014) where a Maximum Likelihood (ML) tree was inferred using the “Phylogenetics Tool”. The resulting tree indicated that investigated accession clusters within the tribe Arabideae. To enhance the analysis, ITS sequences of all available representatives of the Arabideae were retrieved from BrassiBase, aligned with MAFFT v.7.490 using default parameters (Katoh and Standley, 2013), and manually trimmed. The final ML tree was generated using IQ-TREE v.2.2.0 with 10,000 ultrafast bootstrap (UFbootstrap) replicates (Minh et al., 2020). Nodes with UFbootstrap support below 50 were collapsed using Newick Utilities v.1.6.0 (Junier and Zdobnov, 2010). As IQ-TREE produces unrooted trees, *Pseudoturritis turrita*, the basal taxon of the Arabideae, was designated as an outgroup and the tree was rerooted.

Low-coverage genome sequencing of the studied species was performed using the Illumina NovaSeq 6000 platform with 260-bp paired-end reads (3,368,672 × 2 reads; representing *ca*. 2.6× genome coverage; BioProject ID: PRJNA1267952). Adapter sequences and low-quality reads were filtered out using Trimmomatic v.0.39 (Bolger et al., 2014) with the following parameters: ILLUMINACLIP:TruSeq3-PE.fa:2:30:10:2:true, LEADING:10, TRAILING:10, SLIDINGWINDOW:4:20, and MINLEN:40. *De novo* assembly of the plastome was conducted with GetOrganelle v.1.7.7.0 (Jin et al., 2020) using parameters -w 185, –R 15, -k 21,45,65,85,105,127, and the *Scapiarabis saxicola* plastome (MK637807) as the seed. Plastome annotation was performed using the GeSeq v.2.03 pipeline (Tillich et al., 2017) and a graphical visualization was generated with OGDRAW v.1.3.1 (Greiner et al., 2019). The annotated plastome was submitted to GenBank (PV069734; Table S1).

To confirm the phylogenetic placement of the studied species within the Arabideae tribe, the plastid *trnL*-*trnF* region was analyzed. Sequences for this region were sourced from the study by Karl and Koch (2013), and multiple sequence alignments along with ML tree inference were performed using the same methods as for the ITS region. Furthermore, the same phylogenetic pipeline was applied to the available complete plastomes of Arabideae species (Table S1), which were either downloaded from GenBank or obtained from Hendriks et al. (2023). One copy of the inverted repeats (IRs) was removed from the plastome alignment, which was generated using MAFFT, manually reviewed, and trimmed. The final ML tree was inferred using the previously described methods and further refined with phytools v.2.3–0 (Revell, 2024).

### 2.7 Genome size estimation

The genome size was estimated by flow cytometry. The suspension of isolated nuclei was prepared from fully developed intact leaves according to Doležel et al. (2007) and treated by propidium iodide and RNAase (both 50 μg/ml) at room temperature for 5 min and analyzed using a Partec CyFlow cytometer. A fluorescence intensity of at least 5000 particles was recorded. *Carex acutiformis* (1C = 432.8 Mb; Chumová et al., 2021) served as the primary reference (internal) standard.

### 2.8 Repeat identification and annotation

The Illumina data were further utilized to characterize repetitive sequences. For clustering analysis, chloroplast reads were removed using Bowtie2 (v.2.4.5), with the *Scapiarabis saxicola* chloroplast genome sequence (GenBank: MK637807.1) serving as the reference for read mapping. Non-chloroplast reads were filtered for quality, requiring a minimum quality score of 20% and at least 90% of nucleotides above this threshold. The reads were trimmed to a uniform length of 150 bp and interlaced. A subset of reads, representing 0.5× genome coverage (2,214,332 reads at 150 bp), was randomly sampled and used as input for the RepeatExplorer2 pipeline (Novák et al., 2020). Default clustering parameters were applied for species-specific analyses using 64 GB of memory. Superclusters were automatically annotated using the REXdb database (Neumann et al., 2019) and manually verified and corrected. Repetitive sequences were identified and characterized using the RepeatExplorer2 pipeline (Novák et al., 2020) and TAREAN (Novák et al., 2017), utilizing Illumina low-coverage data sampled to 0.5× genome coverage.

To confirm the placement of the studied species within the Arabideae tribe, two comparative clustering analyses were conducted. The first utilized low-coverage Illumina sequencing data, while the second combined Illumina data with Hyb-Seq data.

Comparative analysis with Illumina data: This analysis included sequencing data from four Arabideae species (*Arabis auriculata*, *Ar. cypria*, *Aubrieta canescens* and *Pseudoturritis turrita*; Mandáková et al., 2020b) and publicly available Illumina reads for *Scapiarabis saxicola* (ERR4205135). All reads were trimmed to 100 bp for consistency and sampled to approximately 0.1× genome coverage per species, resulting in 2,660,000 reads. As genome size was unknown for *S. saxicola*, sampling followed the same parameters as for the other studied species. Clustering was performed using RepeatExplorer2 with default settings.

Comparative analysis with Hyb-Seq data: The second analysis combined Illumina sequencing data for the studied species with Hyb-Seq data for *Parryodes axilliflora* (SRR22519503), *Scapiarabis saxicola* (SRR22519334) and *Acirostrum alaschanicum* (SRR22519361) (Hendriks et al., 2023). Reads corresponding to probe sets were filtered by mapping to the Brassicaceae bait sequences from Nikolov et al. (2019) and Angiosperms353 (Johnson et al., 2018) using Bowtie2 (v.2.4.5). All reads were trimmed to 100 bp and randomly sampled to 500,000 reads per species (2,000,000 reads total). Clustering was performed in RepeatExplorer2 using default settings and the comparative clustering option.

Comparative clustering visualizations were generated within the RepeatExplorer2 Galaxy web interface (https://repeatexplorer-elixir.cerit-sc.cz/). Shared tandem repeats were aligned using MAFFT (v.7.490, default settings) and manually adjusted in Geneious Prime (2023).0.1 (https://www.geneious.com).

### 2.9 Oligoprobe design and preparation

In total, five tandem repeats with monomer length ranging from 73 to 454 bp were selected for further investigation (Sat_73, Sat_104, Sat_177, Sat_310 and Sat_454; Table S2). Oligoprobes for these tandem repeats were designed based on reconstructed monomer consensus sequences generated using the TAREAN pipeline. The most conserved regions within the consensus sequences, featuring suitable GC content (30–50%), were manually selected for 60-bp oligoprobes using the Geneious Prime 2023.0.1 software package (https://www.geneious.com). Regions with low potential for self-annealing and hairpin formation were prioritized during the design process. Unmodified synthetic single-stranded DNA (ssDNA) oligonucleotides were dissolved in ultrapure water to a final concentration of 100 μM. Equal molar amounts of complementary oligonucleotides were mixed in a 0.2-ml tube, heated to 95 °C for 5 min in a PCR machine, and immediately transferred to a beaker containing 400 ml of water maintained at 90 °C. The solution was then allowed to cool gradually until the water temperature reached approximately 35 °C, enabling proper annealing of the oligonucleotides. The concentrations of the resulting double-stranded DNA templates were measured using a NanoDrop spectrophotometer. These templates were subsequently labeled using nick translation, as described above.

## 3 Results and discussion

### 3.1 Phylogenetic analysis identified Boechera calcarea *as the member of the Arabideae*

To gain insights into the phylogenetic position of the investigated species, nuclear ITS (ITS1 and ITS2) sequences were analyzed to construct phylogenetic trees. A preliminary large-scale phylogenetic analysis of the Brassicaceae family - including Boechereae and closely related tribes - based on ITS sequences and conducted using BrassiBase (data not shown; Kiefer et al., 2014), suggested that the species belongs to the tribe Arabideae. Subsequently, 349 ITS sequences from Arabideae accessions were realigned and trimmed to a final length of 616 bp. The inferred ML tree revealed that the studied species is sister to the intratribal Arabideae clade comprising *Scapiarabis ariana* and *S. karategina* (UFbootstrap 99%; Fig. S2). Additionally, *Acirostrum alaschanicum* was identified as the sister species to the clade formed by *S. ariana*, *S. karategina* and the studied species with relatively strong support (UFbootstrap 84%) (Fig. S2).

### 3.2 Plastid data confirm the close relationship between species of Scapiarabis, Arcyosperma *and* Parryodes

To investigate the maternal origin of the studied species, the *trnL*-*trnF* region (trimmed alignment of 739 bp) was analyzed, including 311 Arabideae accessions. The resulting ML tree showed that the studied species is sister to a monophyletic clade of all four *Scapiarabis* species (*S. ariana*, *S. karategina*, *S. popovii* and *S. saxicola*; UFbootstrap 100%; Fig. S3). The clade containing *Arcyosperma primulifolium* and *Parryodes axilliflora* was positioned basally to the *Scapiarabis* clade.

We assembled the complete plastome of the studied species and incorporated the available plastomes of 25 Arabideae species to construct a more robust phylogeny. The newly assembled and annotated plastome has a total length of 154,218 bp with a mean GC content of 36.3% and an average base coverage of 351.5 × (Fig. S4). After manual inspection and trimming, the final alignment length was 100,546 bp.

**Fig. 2:**
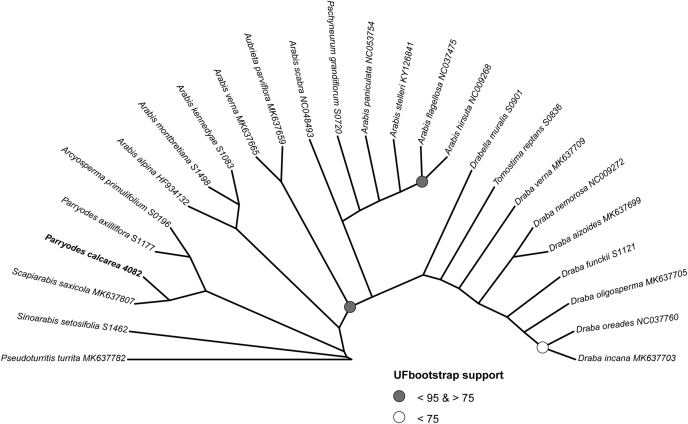
Maximum likelihood tree of the Arabideae tribe based on plastome data. Nodes with ultrafast bootstrap (UF bootstrap) support of 100% are not explicitly labelled for clarity.

The resulting well-supported plastome phylogeny corroborated our earlier findings from single-marker analyses (Fig. 2), indicating that the studied species is closely related to *Scapiarabis saxicola*. The analysis retrieved the two species as closely related to a clade containing *Arcyosperma primulifolium* and *Parryodes axilliflora*.

Our findings are congruent with the most complete Brassicaceae genus-level family phylogenies to date (Hendriks et al., 2023). Their maternal phylogeny placed *Scapiarabis saxicola* as sister to the clade containing *Arcyosperma primulifolium* and *Parryodes axilliflora*, consistent with our results. However, it is important to note that in the nuclear gene tree of Hendriks et al. (2023), *S. saxicola* is positioned as sister to *Acirostrum alaschanicum*, the species absent in their plastome phylogeny. Our attempts to assemble the plastome of *A. alaschanicum* from their Hyb-Seq data (SAMN31015661) were unsuccessful due to low coverage of plastome reads.

### 3.3 Reclassification of Boechera calcarea *as* Parryodes calcarea

Our phylogenetic reconstructions unequivocally demonstrate that *Boechera calcarea* does not belong to the genus *Boechera* or any other genus in the Boechereae (supertribe Camelinodae; German et al., 2023). Instead, our data strongly support its placement in the Arabideae (supertribe Arabodae; German et al., 2023). In this tribe, the species is part of a clade of eight small genera, each comprising one to four species, which are predominantly distributed in the Asian high mountain systems (Koch et al., 2012).

The nuclear ITS phylogeny supports the monophyly of the group to which the former *Boechera calcarea* belongs, yet the chloroplast *trnL-trnF* phylogeny contradicts this, retrieving the species as a distant member of *Scapiarabis*. This incongruence complicates the taxonomic interpretation. While the ITS analysis places *B. calcarea* near *Scapiarabis ariana*, *S. karategina* and *Acirostrum alaschanicum*, the plastid data do not support this topology. Instead, *A. alaschanicum* clusters separately and the group containing *B. calcarea* and *Scapiarabis* forms a distinct lineage.

The observed cytonuclear discordance can be attributed to several factors. As known from other studies on Brassicaceae (e.g., Hendriks et al., 2023; Dominicus et al., 2025), even comprehensive phylogenomic datasets often fail to elucidate the exact causes of such conflicts. The incongruence could be due to incomplete taxon sampling, particularly the absence of *Acirostrum alaschanicum* in the plastid datasets, as its plastome could not be assembled from the available Hyb-Seq data. Second, the cytonuclear discordance may result from incomplete lineage sorting, hybridization, or plastid capture, all of which are plausible in recently diverged or geographically overlapping taxa (Forsythe et al., 2020; Guo et al., 2021; Hendriks et al., 2023). Third, both ITS and *trnL*-*trnF* are relatively short markers (∼600–700 bp) with a low number of informative sites and limited phylogenetic resolution, which may contribute to topological instability (Zhang et al., 2020). Finally, the plastid phylogeny includes only a single representative of *Scapiarabis* (*S. saxicola*), not adequately reflecting the diversity of the genus.

Given these limitations, we adopted a conservative and temporary taxonomic solution that minimizes disruption while reflecting the best-supported evolutionary relationships. Following Chepinoga et al. (2024), we reclassify *Boechera calcarea* as *Parryodes calcarea* (Dudkin) D.A. German & Lysak. This placement distinguishes *P. calcarea*, which has latiseptate fruits and accumbent cotyledons, from *Arcyosperma primulifolium*, which has terete fruits and incumbent cotyledons and occupies a basal position in the ITS-based phylogeny. Moreover, the ITS phylogeny shows that *P. calcarea* is not nested within the existing lineages of *Scapiarabis* or *Acirostrum*, making its placement in *Parryodes* the most parsimonious solution according to current knowledge. However, this does not resolve the broader phylogenetic challenges posed by the unresolved relationships between *Baimashania, Parryodes*, *Pseudodraba*, *Sinoarabis* and other genera within this clade. These genera form a phylogenetically distant clade in the chloroplast phylogeny, underscoring the need for further investigation using broader taxon sampling and genome-wide data.

### 3.4 The correct chromosome number

The chromosome number was determined from root tips and young anthers of ten plants grown from seeds. All plants were confirmed to be diploid with a chromosome number of 2*n* = 16 (Figs. 1E–G, S5 and S6). This finding contradicts the published record of 2*n* = 14 (Doudkin and Volkova, 2013; Fig. S1B). As all chromosome numbers in the genus *Boechera* and tribe Boechereae are derived from the base number *x* = 7, Doudkin and Volkova (2013) based their taxonomic assignment of the species on the morphological and karyological similarity with *Boechera* species and the fact that at least one other species of the genus is native to Russia (*B. falcata*, 2*n* = 14). However, 2*n* = 16 in *Parryodes calcarea* further supports its assignment to the tribe Arabideae.

### 3.5 Cytogenomic analysis confirmed Arabideae-specific chromosomal structures and centromere repositioning

To further analyze the structure of the *Parryodes calcarea* genome, we performed initial comparative chromosome painting on mitotic chromosomes using probes corresponding to differentially labelled BAC contigs of *Arabidopsis thaliana*. These probes were designed to represent selected combinations of genomic blocks specific for Arabideae chromosomes (Mandáková et al., 2020b): Ch1 (genomic blocks A and B), Ch4 (C and Jb), Ch7 (U) and Ch8 (R, Q, and X). This experiment clearly identified Arabideae-specific chromosomal structures in the genome of *P. calcarea* (Fig. S5).

**Fig. 3:**
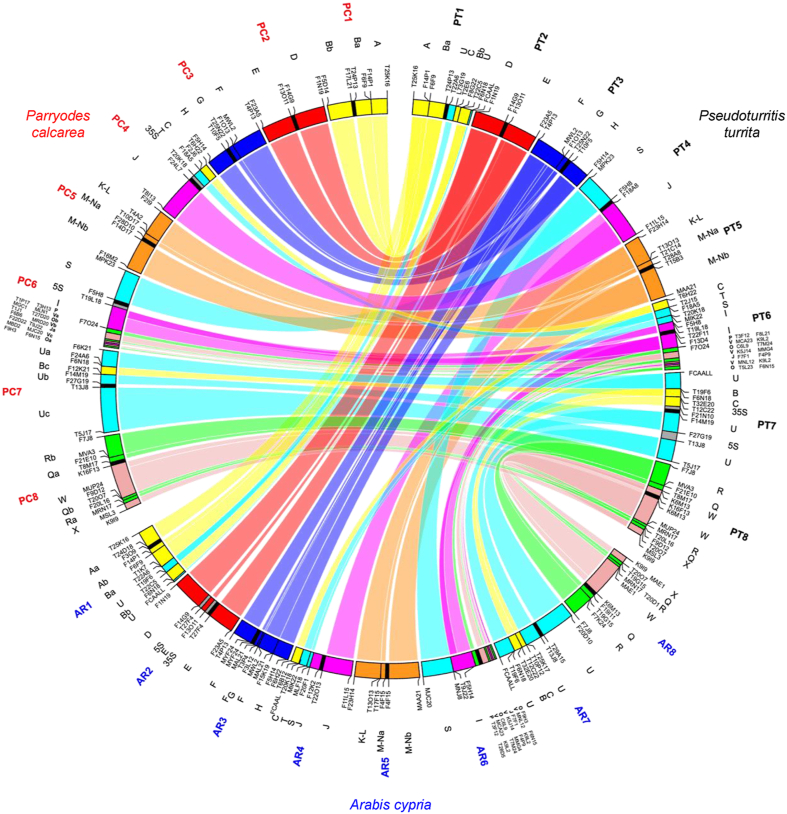
Comparative chromosome structure of *Parryodes calcarea*. A Circos diagram illustrating chromosomal collinearity between *Pseudoturritis turrita* (Mandáková et al., 2020b), *Arabis cypria* (Mandáková et al., 2020b), and *P. calcarea*. Chromosomes are color-coded, with capital letters (A to X) corresponding to the eight chromosomes and 22 genome blocks (GBs) of the Ancestral Crucifer Karyotype (ACK; Mandáková et al., 2019). Black blocks represent centromeres, while grey blocks denote the locations of 35S and 5S rDNA loci. Arabidopsis BAC clones are used as markers for each (sub-)block, enabling determination of collinearity between genomes.

To reconstruct the entire karyotype structure of the species, we used all 22 genomic blocks designed according to the structure of the eight chromosomes of *Pseudoturritis turrita* and evolutionary younger Arabideae clades (Mandáková et al., 2020b; Nowak et al., 2021; Figs. 3, S5 and S7). Comparative chromosome painting revealed that the positions of genomic blocks on all eight chromosomes correspond to those of the younger Arabideae genomes (Arabideae crown group; *Arabis* and *Draba*). Perfect collinearity of genomic blocks was observed for chromosomes Pc1, Pc2, Pc3, Pc4, Pc5, and Pc8 (Fig. 1, Fig. 2 and S7). On chromosome Pc7, we identified a short fragment of genomic block B within U (Fig. 1H and I). Our unpublished data suggest that this feature is common in all Arabideae species but was not detected in previous studies due to the lower resolution of earlier chromosome painting experiments (Mandáková et al., 2020b; Nowak et al., 2021). The B-block fragment within the larger block U on chromosome Pc7 is more ancestral than the origin of the Arabideae (Liu et al., 2024). Chromosome Pc6 exhibited a complex shuffling of genomic blocks, resulting in two separate parts of block O and three parts of block V (Fig. 1H and I). Although the structure of Pc6 slightly differs from the structure of chromosome 6 homeologs in other Arabideae genomes, we attribute these differences to the higher resolution of chromosome painting compared to earlier studies (Figs. 3 and S7).

A positional shift of centromeres has already been found on five out of six chromosomes in the Arabideae crown-group (Mandáková et al., 2020b). Therefore, a precise characterization of the centromere positions was performed using a BAC-by-BAC approach. Chromosomes Pc1, Pc2 and Pc3 exhibited ancestral-like centromere positions as in *Pseudoturritis turrita*. In contrast, the centromeres of chromosomes Pc5, Pc6, Pc7 and Pc8 showed independent positional shifts compared to the homeologous centromeres in *P. turrita* as well as in Arabideae crown-group species. The structure of chromosome Pc4 matches that of the Arabideae crown-group, which differs from *P. turrita* by a reciprocal translocation. Remarkably, no centromere shifts were observed on this chromosome within the Arabideae crown group (Figs. 1H, I and 2).

Our chromosome painting data provide robust evidence for the placement of *Parryodes calcarea* to the Arabideae. The shared chromosomal signatures with both the ancestral genome of *Pseudoturritis turrita* and the species of the Arabideae crown group confirm that the taxon represents an intermediate transition between the ancestral and crown-group intratribal clades. Additionally, our findings support previous observations that centromere repositioning serves as the predominant mechanism of chromosome differentiation in the tribe Arabideae, a phenomenon that is otherwise rare in the Brassicaceae (Mandáková et al., 2020b). While the evolutionary triggers of centromere mobility remain unclear, both adaptive and neutral effects have been proposed. For example, the centromere shift could influence chromosome morphology and recombination frequency as well as gene expression within the epigenetically silenced (peri)centromeric heterochromatin. The conserved synteny of homeologous chromosomes, but divergent centromere position can serve as a post-zygotic reproductive barrier due to chromosomal mispairing in hybrids (Bracewell et al., 2019; Mandáková et al., 2020b; Ansai et al., 2024). However, in *P. calcarea* and other Arabideae species, it is difficult to determine the adaptive significance of the observed centromere repositioning events without sister species comparisons, population-level data and functional analyzes. Further studies integrating epigenomic, transcriptomic and recombination data will be necessary to assess the evolutionary consequences of centromere mobility in the Arabideae.

### 3.6 Repeatome dynamics and chromosomal localization of repeats in Parryodes calcarea

Genome size of the investigated species was estimated by flow cytometry to be 664 Mb/1C (Fig. S8A). Repeats were found to constitute 64.87% of the *Parryodes calcarea* genome (Table S3). The repeatome composition of *P. calcarea*, dominated by LTR retrotransposons - particularly Ty3/Gypsy elements - is consistent with patterns observed in other Arabideae and Brassicaceae clades (e.g., Zhang et al., 2020; Hloušková et al., 2019). Tandem repeats constituted 0.40% of the genome (Table S3). Five distinct tandem repeat sequences were identified, with monomer lengths ranging from 73 to 454 bp. The most abundant tandem repeat, Sat_105 (105 bp), accounted for 0.2% of the genome, followed by Sat_310 (310 bp; 0.09%) and Sat_177 (177 bp; 0.07%). The remaining tandem repeats, Sat_454 and Sat_73, were present at lower proportions (0.02% and 0.01%, respectively). Notably, tandem repeat Sat_177 exhibited a pairwise sequence identity of 74% with another cluster identified as a tandem repeat with a monomer size of 176 bp (0.01%; Sat_177_v2), suggesting these are likely two variants of a tandem repeat family.

To further elucidate the karyotype structure of the studied accession, we conducted chromosome localization of the identified tandem repeats. Arabidopsis-like telomeric repeat (TTTAGGG)_*n*_ and species-specific subtelomeric satellite Sat_177 were exclusively detected at the chromosome termini, confirming the absence of interstitial telomeric repeats (Fig. 1G and S6). In contrast, remaining four identified tandem repeats were localized within the heterochromatic pericentromeric regions. Specifically, Sat_73 and Sat_105 were detected in the pericentromere of chromosome Pc3, while the pericentromere of chromosome Pc4 contained 35S rDNA and Sat_105. Chromosome Pc6 displayed 5S rDNA and Sat_310 in its pericentromeric region, whereas Sat_310 alone was identified in the pericentromere of chromosome Pc7. The pericentromere of chromosome Pc8 harbored Sat_454 (Figs. 1F, G and S6). Notably, no tandem repeats were found in the pericentromeres of chromosomes Pc1, Pc2 and Pc5. Interestingly, no pericentromeric satellite was shared among all chromosomes. Instead, four chromosome-specific tandem repeats were observed, suggesting rapid centromere evolution in this species. This pattern aligns with the hypothesis of accelerated centromere evolution in the tribe Arabideae (Mandáková et al., 2020b). To compare the repetitive sequences in the genome of *Parryodes calcarea* with those of other Arabideae species (*Acirostrum alaschanicum*, *Arabis auriculata*, *Ar. cypria*, *Aubrieta canescens*, *Parryodes axilliflora, Pseudoturritis turrita* and *Scapiarabis saxicola*), two comparative clustering analyses were conducted using the RepeatExplorer2 pipeline. The first comparative clustering analysis, based on low-coverage Illumina data (Fig. S8A), demonstrated that *P. calcarea* and *S. saxicola* have highly similar repeat profiles, with notable differences in the abundance of specific repeat types. The second comparative clustering analysis (Fig. S8B) incorporated Hyb-Seq data (Hendriks et al., 2023) alongside low-coverage Illumina sequencing data of *P. calcarea*. This analysis revealed uneven representation between the two datasets: Hyb-Seq data provided high coverage of target regions, whereas low-coverage sequencing better captured non-target regions. Tandem repeats Sat_310 and Sat_177 were detected in all species. The absence of Sat_105 in some species is likely attributable to the use of Hyb-Seq data, which tends to underrepresent highly repetitive sequences. However, this absence may also reflect genuine evolutionary processes, such as lineage-specific repeat loss or replacement. In contrast, Sat_177 was consistently detected across all species, albeit with two distinct variants, suggesting ongoing diversification of this repeat family. These patterns are consistent with the dynamic evolution of satellite DNA in the Brassicaceae and other angiosperm lineages.

The absence of shared pericentromeric satellites across all chromosomes, combined with the presence of multiple unique repeat families, supports the hypothesis of dynamic centromere evolution in Arabideae (Mandáková et al., 2020b).

## 4 Conclusions

This study reclassified *Boechera calcarea* as *Parryodes calcarea*, a distinct species of one of the more ancestral clades in the tribe Arabideae (Brassicaceae). Comprehensive cytogenetic analyses revealed a diploid chromosome number of 2*n* = 16 and Arabideae-specific chromosomal signatures, including several centromere repositioning events. Excluding *P. calcarea* from the Boechereae left *B. falcata* as the only extralimital *Boechera* species in Asia. These findings emphasize the value of integrative approaches to resolve taxonomic ambiguities and improve our understanding of the diversification of the Brassicaceae in Asia.

## CRediT authorship contribution statement

**Terezie Mandáková:** Writing – review & editing, Writing – original draft, Visualization, Supervision, Investigation, Funding acquisition, Conceptualization. **Milan Pouch:** Writing – original draft, Visualization, Investigation, Data curation, Conceptualization. **Petra Hloušková:** Writing – original draft, Investigation, Data curation. **Dmitry A. German:** Writing – original draft, Validation, Investigation. **Pavel Trávníček:** Investigation. **Michael D. Windham:** Writing – original draft, Validation. **Martin A. Lysak:** Writing – review & editing, Supervision, Funding acquisition, Conceptualization.

## Declaration of competing interest

The authors declare that they have no known competing financial interests or personal relationships that could have appeared to influence the work reported in this paper.
