# Supplementary material for: Boechera or not? Genomic insights and taxonomic reassessment of the misclassified Asian species B. calcarea (Brassicaceae)
Source: Plant Divers. 2025 Jul 10;48(1):107–16. doi: 10.1016/j.pld.2025.06.009 (PMC12918183; doi:10.1016/j.pld.2025.06.009)
Supplement: Multimedia component 1 [file mmc1.pdf]

A

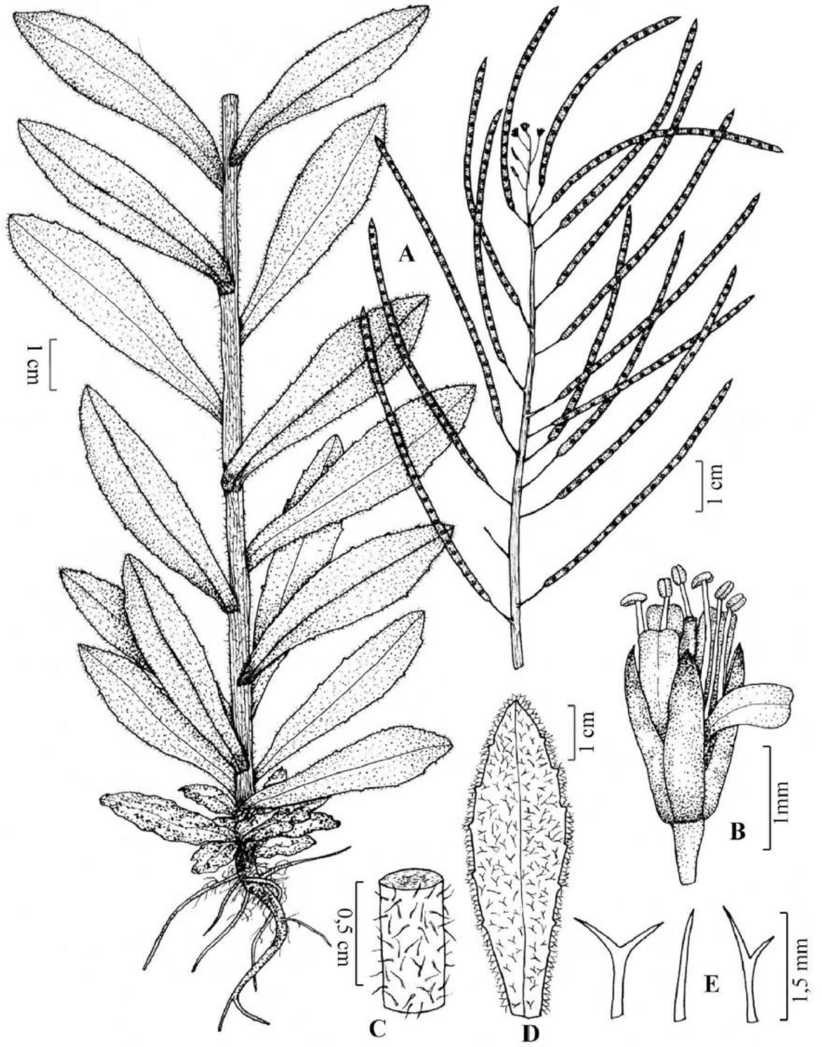

Figure 2. *Boechera calcarea* Dudkin. —A. Fertile plant showing the basal rosette of leaves present at anthesis. —B. Flower. —C. Basal portion of stem. — D. Stem leaf blade. —E. Representative trichomes from stem and leaf. Drawn from the type *R. V. Doudkin s.n.* (LE).

B

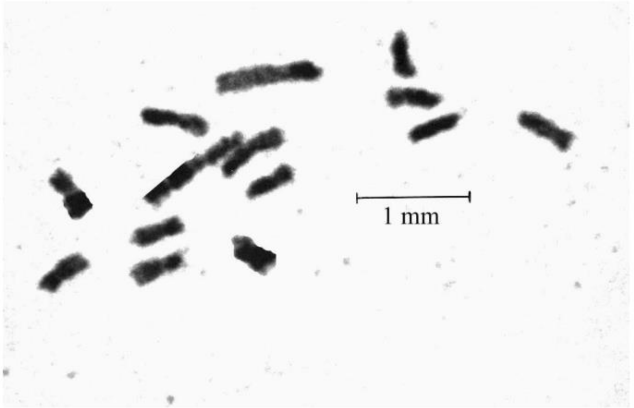

Figure 1. *Boechera calcarea* Dudkin. Chromatids at meiotic metaphase, $2n = 14$. Taken from the paratype from Mt. Sestra, limestone rock near summit, 16 July 2008, *R. V. Doudkin s.n.* (BGI).

Suppl. Fig. 1 Botanical drawing (A) and chromosome figure (B) of *Boechera calcarea* from Doudkin and Volkova (2013).

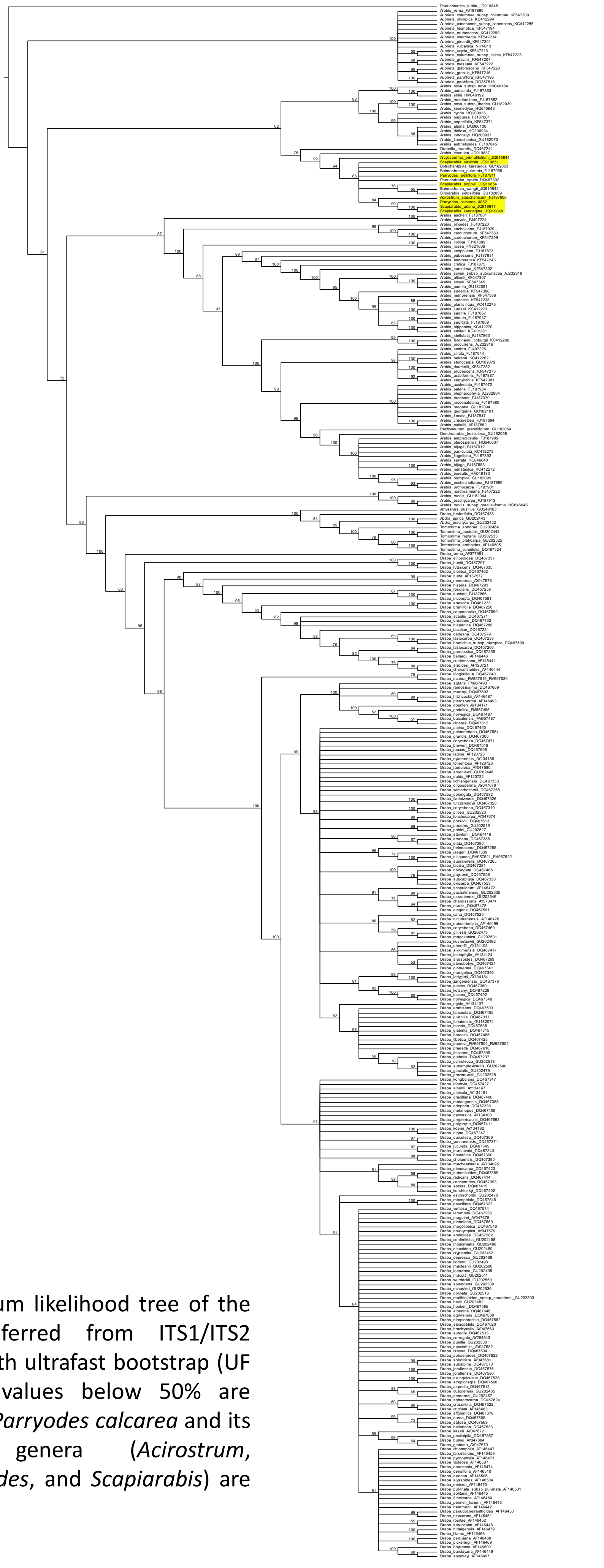

Suppl. Fig. 2 Maximum likelihood tree of the tribe Arabideae inferred from ITS1/ITS2 sequences. Nodes with ultrafast bootstrap (UF bootstrap) support values below 50% are collapsed for clarity. *Parryodes calcarea* and its closely related genera (*Acirostrum*, *Arcyosperma*, *Parryodes*, and *Scapiarabis*) are highlighted in yellow.

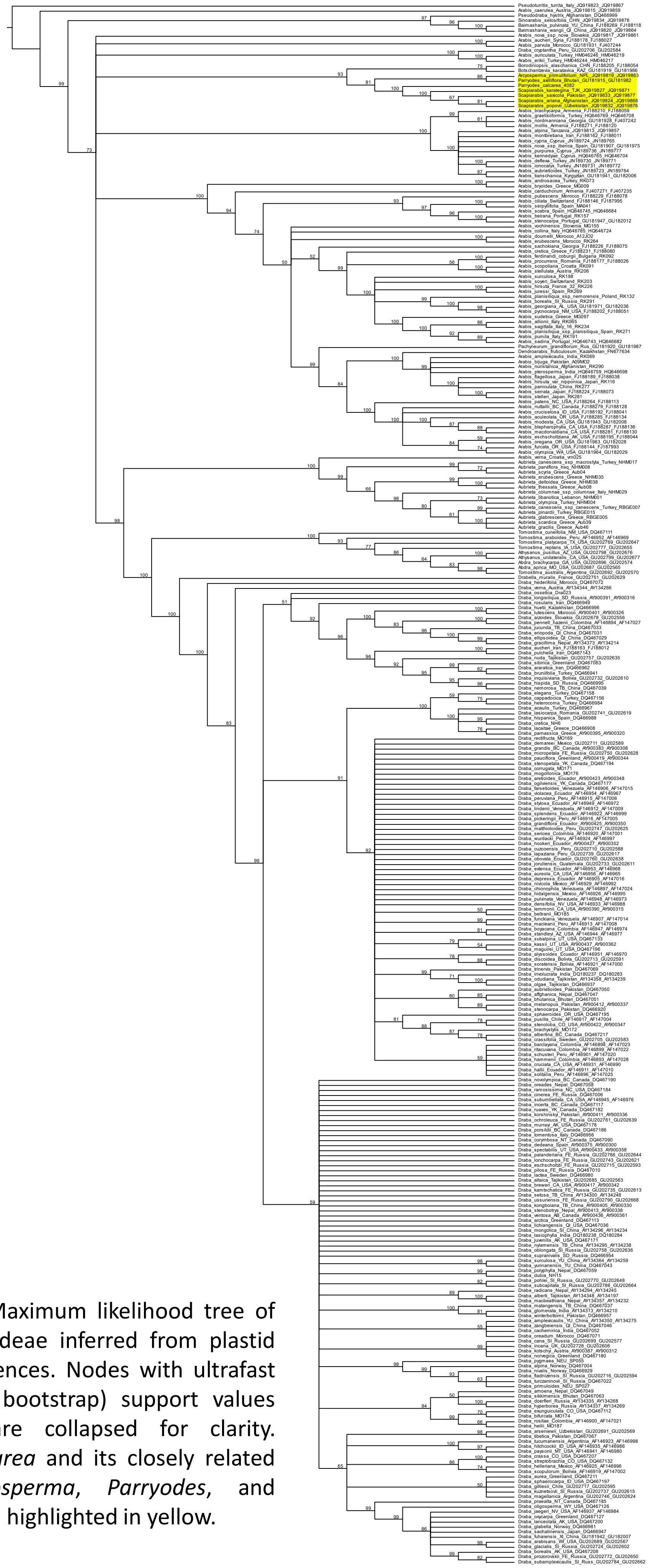

Suppl. Fig. 3 Maximum likelihood tree of the tribe Arabideae inferred from plastid trnL–trnF sequences. Nodes with ultrafast bootstrap (UF bootstrap) support values below 50% are collapsed for clarity. *Parryodes calcarea* and its closely related genera (*Arcyosperma*, *Parryodes*, and *Scapiarabis*) are highlighted in yellow.

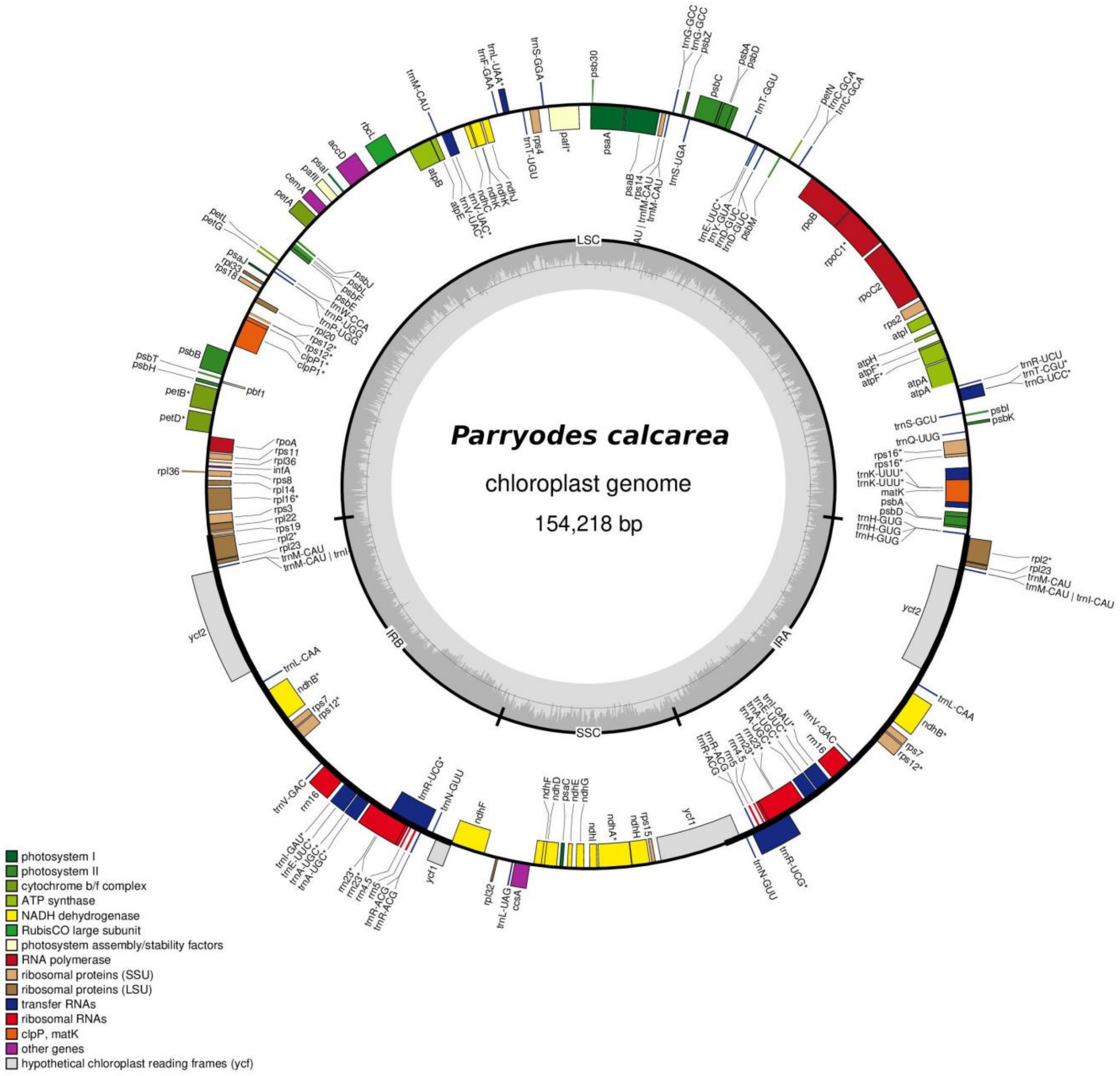

Suppl. Fig. 4 Newly assembled and annotated plastome of *Parryodes calcarea*.

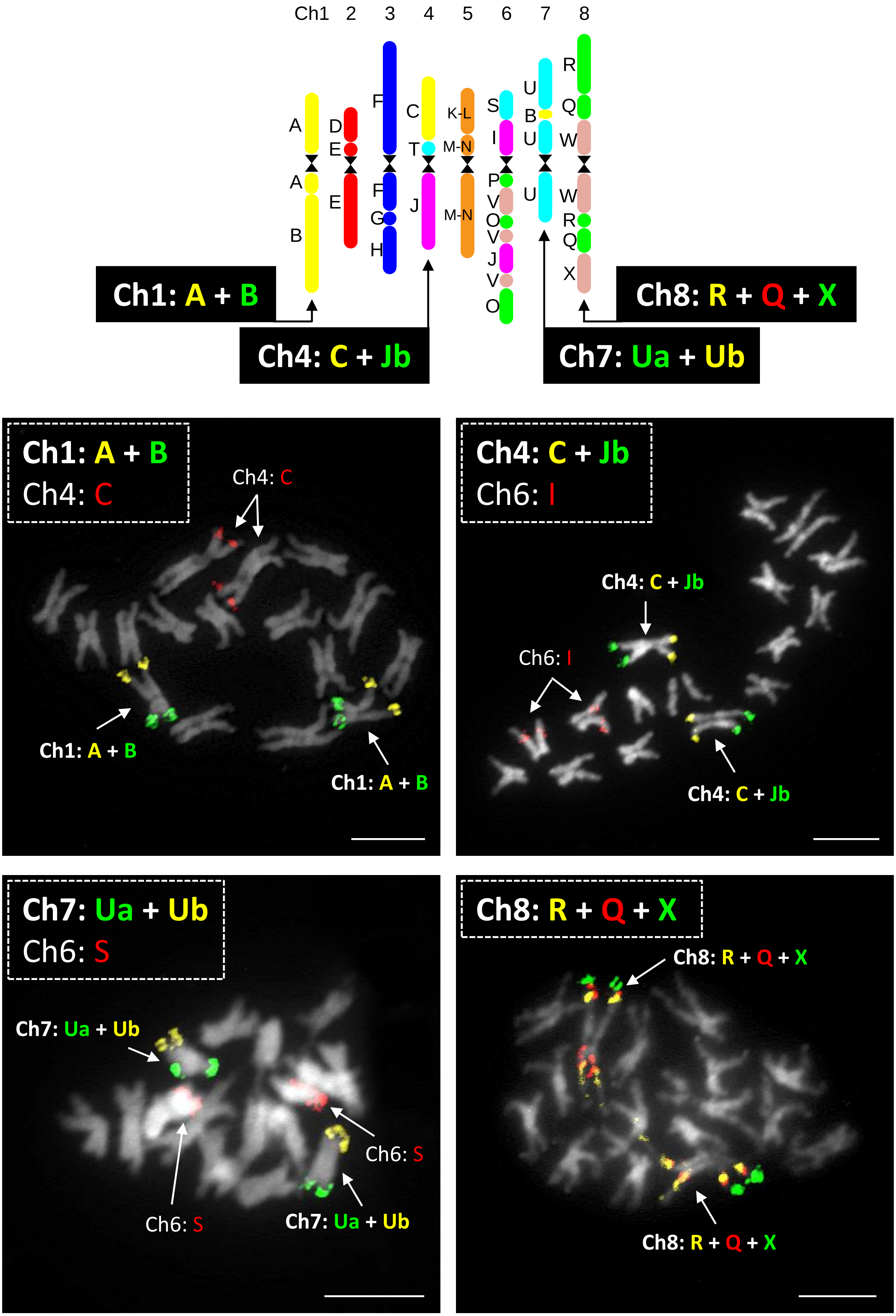

Suppl. Fig. 5 Comparative chromosome painting of mitotic chromosomes of *Parryodes calcarea*, prepared from root tips and hybridized with Arabidopsis BAC clone pools grouped into contigs representing marker genomic block combinations of Arabideae crown-group species (Mandáková et al., 2020b). Chromosomes were counterstained with DAPI. Scale bars = 10 µm.

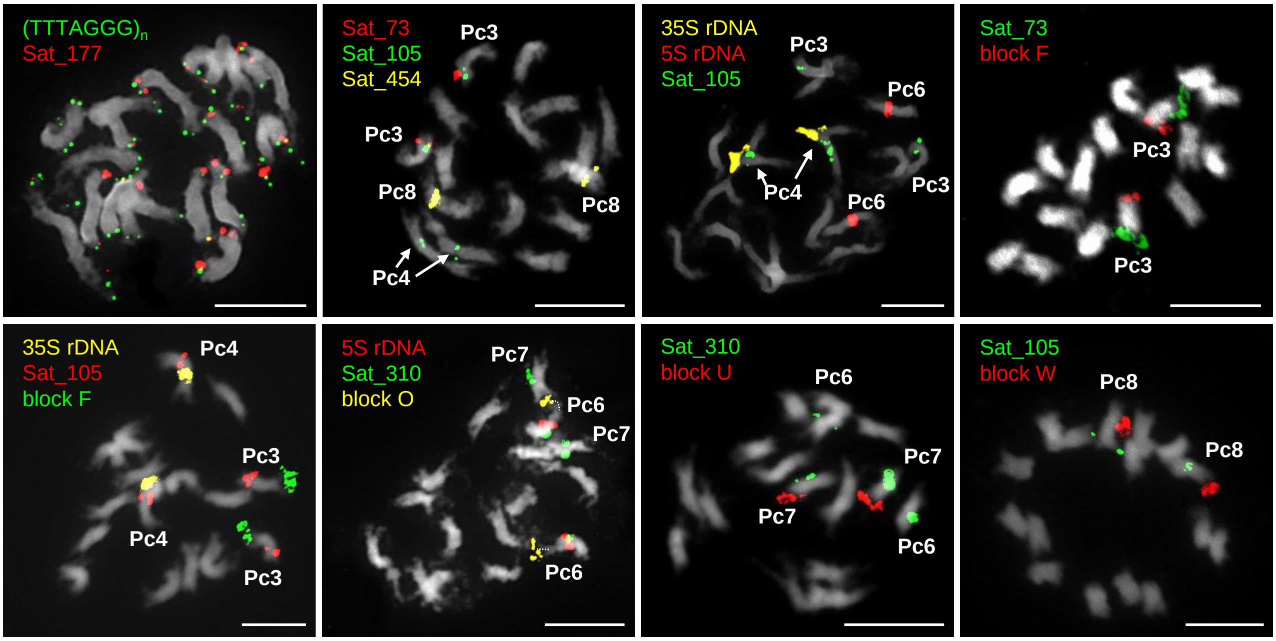

Suppl. Fig. 6 Chromosome localization of the selected tandem repeats on mitotic metaphase chromosomes of *Parryodes calcarea*. Chromosomes were counterstained by DAPI; FISH signals are shown in colour as indicated. Detailed information on the localized repeats is provided in Suppl. Tab. 2. Scale bars, 10 µm.

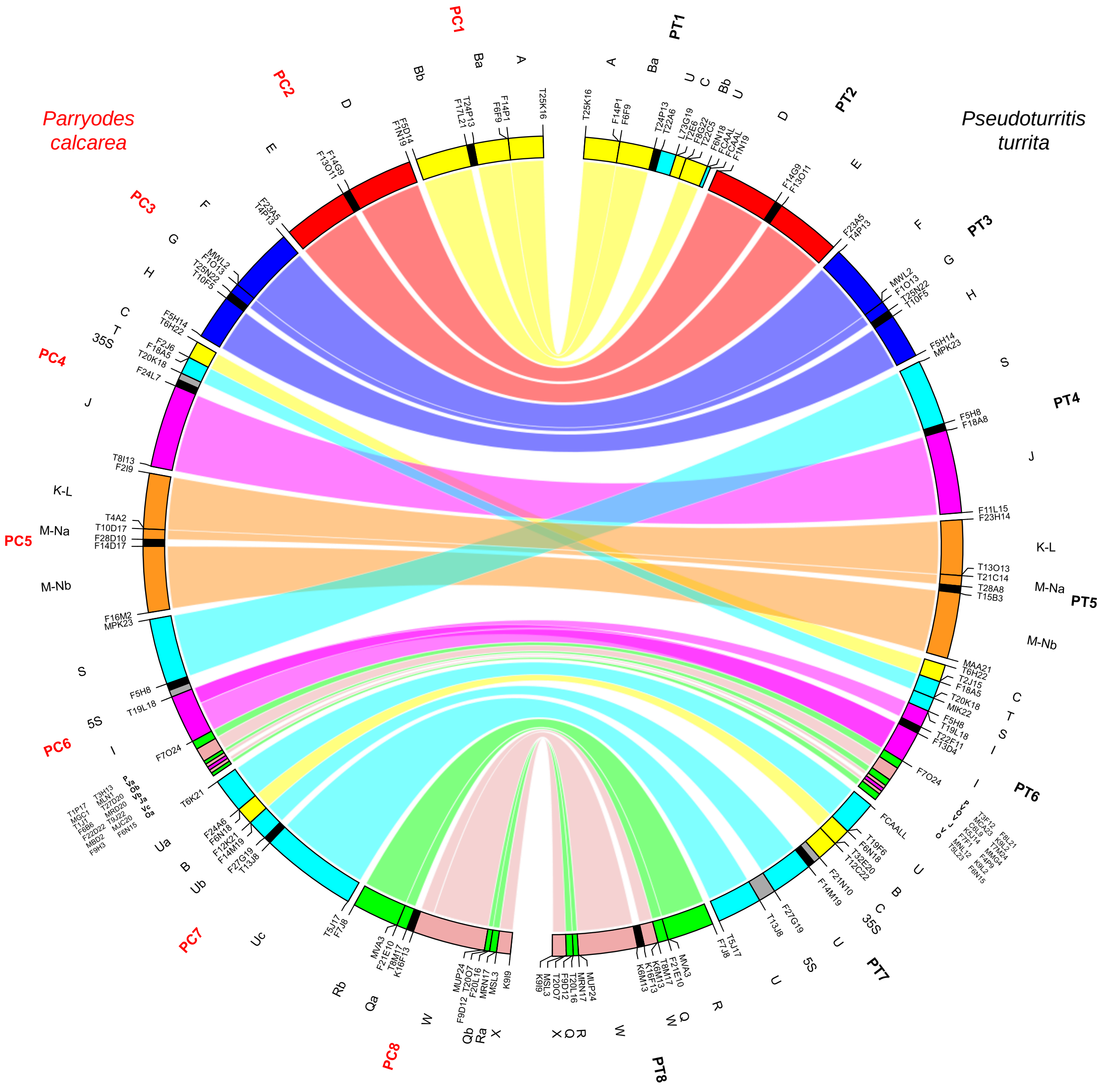

Suppl. Fig. 7 Comparative chromosome structure of *Parryodes calcarea*. A Circos diagram illustrating chromosomal collinearity between *Pseudoturritis turrita* (Mandáková et al., 2020b) and *Parryodes calcarea*. Chromosomes are color-coded, with capital letters (A to X) representing the eight chromosomes and 22 genome blocks (GBs) of the Ancestral Crucifer Karyotype (ACK; Mandáková et al., 2019). Black blocks indicate centromeres, and grey blocks mark the positions of 35S and 5S rDNA loci. Arabidopsis BAC clones serve as markers for each (sub-)block, facilitating the comparison of collinearity between genomes.

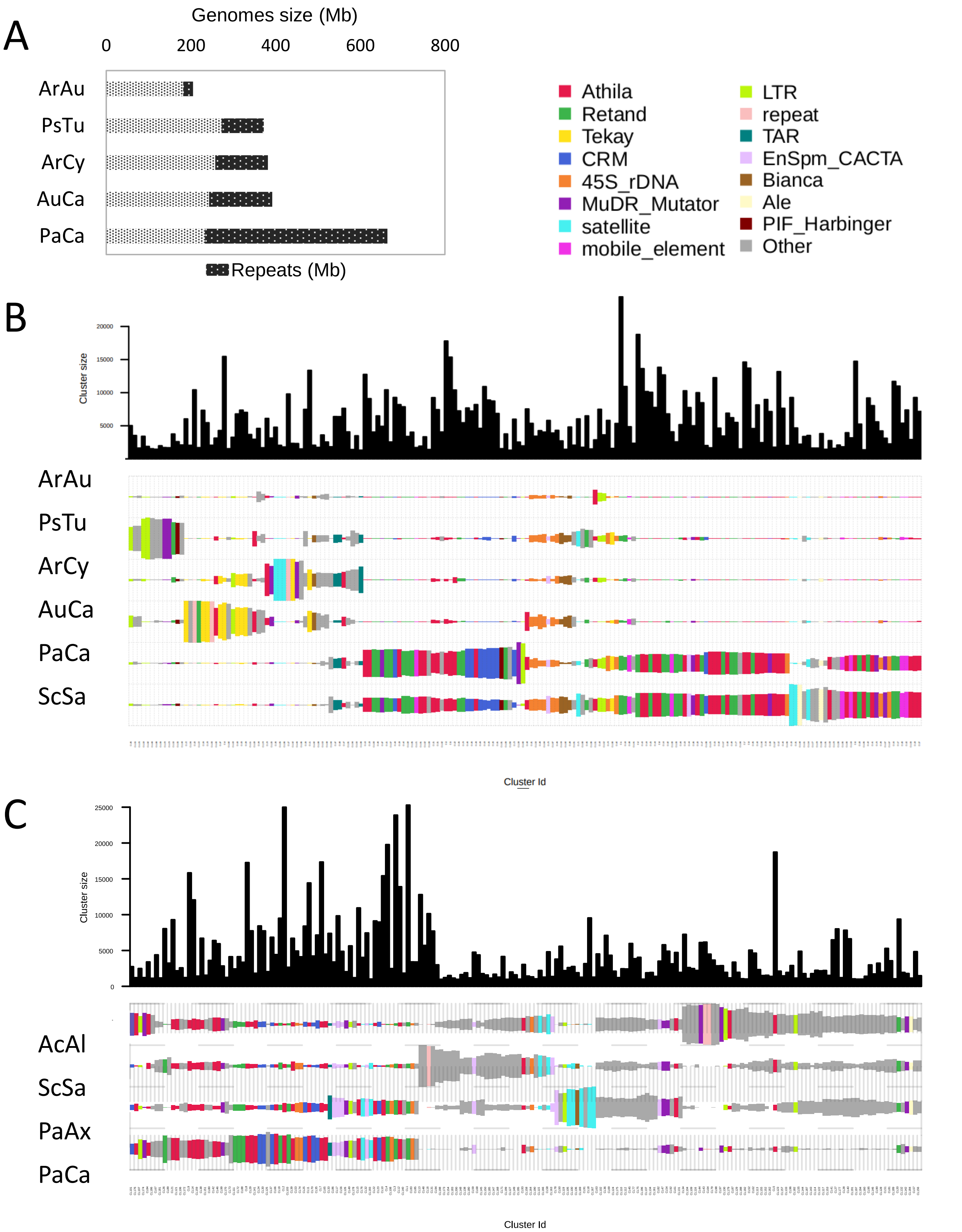

Suppl. Fig. 8 Comparative clustering analysis of repetitive sequences in selected species. (A) Genome sizes and the proportions of identified repetitive sequences for each species, based on data from Mandáková et al. (2020b) and *Parryodes calcarea*. (B) Analysis of *Arabis auriculata*, *Arabis cypria*, *Aubrieta canescens*, *Parryodes calcarea*, *Pseudoturritis turrita*, and *Scapiarabis saxicola*. (C) Analysis of *Acirostrum alaschanicum*, *Parryodes axilliflora*, *Parryodes calcarea*, and *Scapiarabis saxicola*. The sequence composition of the 200 most abundant clusters is displayed, with the size of each rectangle proportional to the genome proportion of a cluster for each species (*Acirostrum alaschanicum*: AcAl, *Arabis auriculata*: ArAu, *Arabis cypria*: ArCy, *Aubrieta canescens*: AuCa, *Parryodes axilliflora*: PaAx, *Parryodes calcarea*: PaCa, *Pseudoturritis turrita*: PsTu, *Scapiarabis saxicola*: ScSa). The bar plot in the top row indicates cluster sizes based on the number of reads in the comparative analysis.

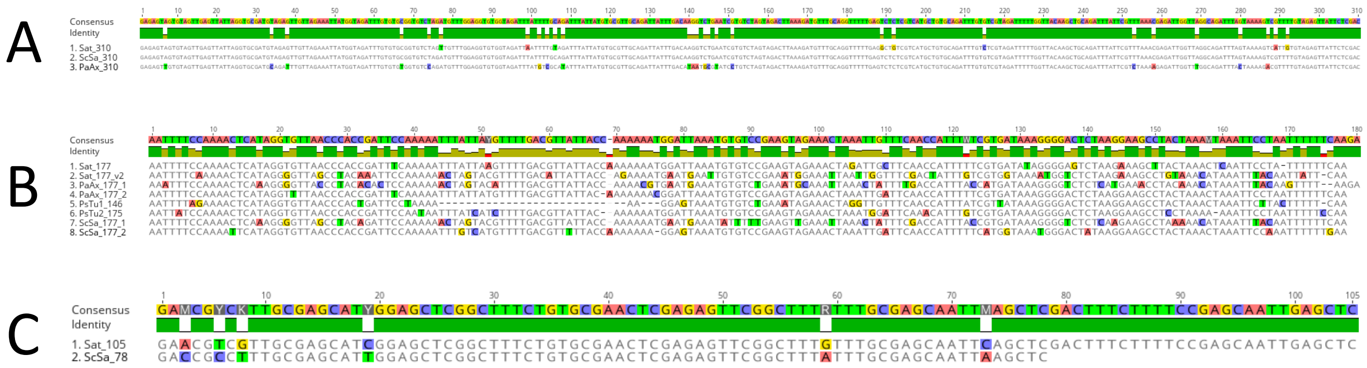

Suppl. Fig. 9 Multiple sequence alignments of shared tandem repeats across species. Comparisons of tandem repeats between *Parryodes calcarea* (Sat_310, Sat_177, and Sat_105), *Parryodes axilliflora* (PaAx), *Pseudoturritis turrita* (PsTu), and *Scapiarabis saxicola* (ScSa). (A) Alignment of Sat_310. (B) Alignment of Sat_177. (C) Alignment of Sat_105.
