# Supplementary material for: Boechera or not? Genomic insights and taxonomic reassessment of the misclassified Asian species B. calcarea (Brassicaceae)
Source: Plant Divers. 2025 Jul 10;48(1):107–16. doi: 10.1016/j.pld.2025.06.009 (PMC12918183; doi:10.1016/j.pld.2025.06.009)
Supplement: Multimedia component 2 [file mmc2.docx]

**SUPPLEMENTARY INFORMATION**

**Suppl. Tab. 1** List of Arabideae species with assembled plastomes used in this study.

| **Species** | **ENA/GenBank code or Dryad doi published sequences** | **SRA code/BioProject** | **NBC DNA bank**  **accession** | **Source** |
| --- | --- | --- | --- | --- |
| *Parryodes calcarea* | PC069734 |  |  | this study |
| *Arabis alpina* | HF934132 |  |  | Melodelima and Lobréaux (2013) |
| *Arabis stelleri* | KY126841 |  |  | Raman et al. (2017) |
| *Aubrieta parviflora* | MK637659 | PRJEB38700 |  | Walden et al. (2020) |
| *Arabis verna* | MK637665 | PRJEB38700 |  | Walden et al. (2020) |
| *Draba aizoides* | MK637699 | PRJEB38700 |  | Walden et al. (2020) |
| *Draba incana* | MK637703 | PRJEB38700 |  | Walden et al. (2020) |
| *Draba oligosperma* | MK637705 | PRJEB38700 |  | Walden et al. (2020) |
| *Draba verna* | MK637709 | PRJEB38700 |  | Walden et al. (2020) |
| *Pseudoturritis turrita* | MK637782 | PRJEB38700 |  | Walden et al. (2020) |
| *Scapiarabis saxicola* | MK637807 | PRJEB38700 |  | Walden et al. (2020) |
| *Arabis hirsuta* | NC_009268 |  |  |  |
| *Draba nemorosa* | NC_009272 |  |  |  |
| *Arabis flagellosa* | NC_037475 |  |  |  |
| *Draba oreades* | NC_037760 |  |  |  |
| *Arabis scabra* | NC_048493 |  |  |  |
| *Arabis paniculata* | NC_053754 |  |  |  |
| *Arcyosperma primulifolium* | S0196sl | SAMN31015672 | 4012502100 | Hendriks et al. (2023) |
| *Pachyneurum grandiflorum* | S0720 | SAMN31015686 | 4032059836 | Hendriks et al. (2023) |
| *Tomostima reptans* | S0836 | SAMN31015744 | 4032059731 | Hendriks et al. (2023) |
| *Drabella muralis* | S0901 | SAMN30711618 | 4032059780 | Hendriks et al. (2023) |
| *Arabis kennedyae* | S1083 | SAMN31015610 | 4032059594 | Hendriks et al. (2023) |
| *Draba funckii* | S1121 | SAMN30711482 | 4032059444 | Hendriks et al. (2023) |
| *Parryodes axilliflora* | S1177 | SAMN30711528 | 4032059523 | Hendriks et al. (2023) |
| *Sinoarabis setosifolia* | S1462 | SAMN30711657 | 4057164593 | Hendriks et al. (2023) |
| *Arabis montbretiana* | S1498 | SAMN30711476 | 4057163481 | Hendriks et al. (2023) |

**Suppl. Tab. 2** Oligonucleotide probes specific to tandem repeats of Parryodes calcarea.

| **Tandem repeat** | **Monomer**  **(bp)** | **Genome**  **proportion (%)** | **Probe sequence** |
| --- | --- | --- | --- |
| Sat_73 | 73 | 0.01 | GCATCAAACTGAAAATAAGTCTGCAATAACTGATACAAAAGTCTACAGTCTGAAACAAAT |
| Sat_105 | 105 | 0.20 | CGAGCAATTGAGCTCGAACGTCGTTGCGAGCATCGGAGCTCGGCTTTCTGTGCGAACTCG |
| Sat_177 | 177 | 0.07 | GTAAGCTTTCTTAGACTCCCCTATATCACGAAAAATGGTTGAAGCAATCTAGTTTCTACT |
| Sat_310 | 310 | 0.09 | GCGATGTAGAGTTGTTAGAAATTATGGTAGATTTGTGTGCGGTGTCTAGTTGTTTGGAGG |
| Sat_454 | 454 | 0.02 | GTAAGATATAGTTTACAATTTAAGATTTAGAGTTTATGATTAAGGGTTAGAGATTTAGGG |

**Suppl. Tab. 3** Proportions (%) of repetitive sequences in Parryodes calcarea genome.

| LTR retroelements | All (including LTR retroelements of unknown superfamily) | | | 52.63 |
| --- | --- | --- | --- | --- |
|  | Ty3/gypsy | All | | 49.11 |
|  |  | Unclassified | | 0.31 |
|  |  | Athila | | 24.92 |
|  |  | Chromovirus | All | 7.59 |
|  |  |  | Tekay | 0.29 |
|  |  |  | CRM | 7.30 |
|  |  | Retand | | 16.29 |
|  | Ty1/copia | All | | 1.19 |
|  |  | Unclassified | | 0.00 |
|  |  | Ale | | 0.15 |
|  |  | Bianca | | 0.01 |
|  |  | Ikeros | | 0.21 |
|  |  | Ivana | | 0.02 |
|  |  | SIRE | | 0.20 |
|  |  | TAR | | 0.53 |
|  |  | Tork | | 0.07 |
| DNA transposons | All (including unclassified DNA transposons) | | | 6.43 |
|  | CACTA | | | 3.42 |
|  | MuDR | | | 2.67 |
|  | hAT | | | 0.01 |
|  | Harbinger | | | 0.15 |
|  | Helitron | | | 0.00 |
| LINEs | | | | 0.52 |
| rDNA | | | | 1.80 |
| Tandem repeats | | | | 0.40 |
| Unclassified repeats | | | | 3.10 |
| Low/single-copy sequences | | | | 35.12 |
| **Repeats** | | | | **64.88** |
